# Single Isolated Sacral Metastasis From Clear Cell Renal Cell Carcinoma: A Case Report

**DOI:** 10.1155/crom/5545096

**Published:** 2025-09-11

**Authors:** Salah Ahmed Binziad, Mohammed Abdulla Baamer, Waleed Khaled Kaleem, Moawia Mohammed Elhassan

**Affiliations:** ^1^General and Neurosurgery Department, Hadhramout University College of Medicine (HUCOM), Mukalla, Hadhramaut, Yemen; ^2^Department of Medicine, Hadhramout University College Medicine (HUCOM), Mukalla, Hadhramaut, Yemen; ^3^Mukalla Radiotherapy Center, Mukalla, Hadhramaut, Yemen

**Keywords:** clear cell renal cell carcinoma, multidisciplinary management, sacral metastasis, stereotactic body radiotherapy, sunitinib

## Abstract

This case report describes a rare occurrence of a single isolated sacral metastasis in a 59-year-old male with clear cell renal cell carcinoma (ccRCC). The patient presented with progressive lower back pain and sacral numbness following a fall. Initial imaging revealed a destructive lytic lesion in the S1–S2 region and a left renal mass. A CT-guided biopsy of the sacral lesion confirmed metastatic ccRCC. PET-CT confirmed no additional metastases. Multidisciplinary management included partial left nephrectomy for the primary renal tumor, stereotactic body radiotherapy (SBRT; 45 Gy/15 fractions) targeting the sacral metastasis, and immunotherapy with sunitinib. The patient experienced significant pain relief within 1-week post radiotherapy and demonstrated stable disease with near-resolution of the sacral lesion at 3-month follow-up. At 12 months, imaging confirmed sustained disease stability. This case highlights the importance of considering metastatic RCC in patients with isolated sacral lesions, even in the absence of disseminated disease, and underscores the efficacy of combining localized ablation, radiotherapy, and targeted systemic therapy. Early diagnosis and a tailored multidisciplinary approach are critical for optimizing outcomes in such rare presentations.

## 1. Introduction

Renal cell carcinoma (RCC), the most common malignancy of the kidney, is notorious for its propensity to metastasize to distant sites, with the lungs, liver, and bones being the most frequently involved organs [1]. Clear cell renal cell carcinoma (ccRCC), the predominant histologic subtype, accounts for 70%–80% of cases and demonstrates a similar metastatic pattern, often with a predilection for osteolytic bone lesions [2]. Bone metastases from RCC typically affect the axial skeleton, including the spine, pelvis, and long bones, and are associated with significant morbidity due to pain, pathologic fractures, and neurologic compromise [1]. However, isolated sacral metastases are exceedingly rare, representing less than 1% of reported RCC metastases, and pose unique diagnostic and therapeutic challenges [3].

The rarity of sacral involvement often leads to delayed diagnosis, as symptoms such as lower back pain or sacral numbness may initially be attributed to trauma or degenerative conditions, particularly in the absence of a known primary malignancy [3]. This diagnostic ambiguity underscores the importance of maintaining a high index of suspicion for metastatic disease in patients with a history of RCC or atypical presentations. Advanced imaging modalities, including MRI, CT, and PET-CT, play a pivotal role in identifying primary tumors and metastatic lesions, while histopathologic confirmation remains the gold standard for definitive diagnosis [4].

The management of metastatic RCC has evolved significantly with the advent of targeted therapies, such as tyrosine kinase inhibitors (TKIs), and precision radiotherapy techniques like stereotactic body radiotherapy (SBRT). Multidisciplinary approaches integrating surgery, radiation, and systemic therapy are increasingly recognized as critical to optimizing outcomes, particularly for oligometastatic disease [5]. However, evidence guiding the management of isolated sacral metastases remains sparse, given their rarity. We detail the diagnostic pathway, emphasizing the role of advanced imaging, and highlight the successful application of a multimodal treatment strategy combining partial nephrectomy, SBRT, and sunitinib. By presenting this rare clinical scenario, we aim to reinforce the importance of considering metastatic RCC in sacral lesions, advocate for timely multidisciplinary intervention, and contribute to the limited literature on this uncommon manifestation of ccRCC.

## 2. Case Presentation

A 59-year-old male from Hadhramout, Yemen, had a history of falling down on his back since 1 month ago and presented of progressive lower back pain and sacral numbness. His physical exam revealed tenderness and swelling over the sacrum and diminished perianal sensation.

### 2.1. Imaging

**Figure 1:**
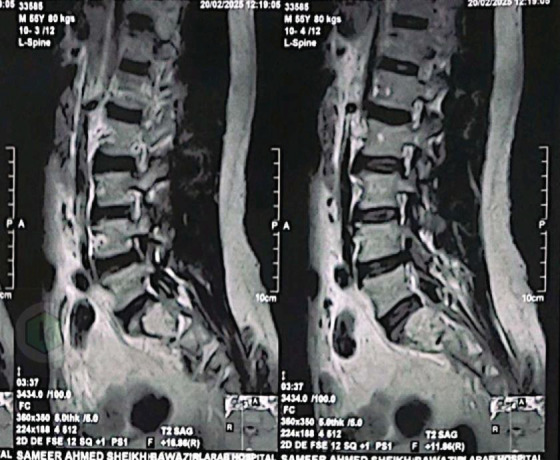
Sagittal T1-weighted MRI showing osteolytic sacral lesion (S1–S2).

MRI demonstrated a destructive lytic lesion in the S1–S2 region (Figure 1).

**Figure 2:**
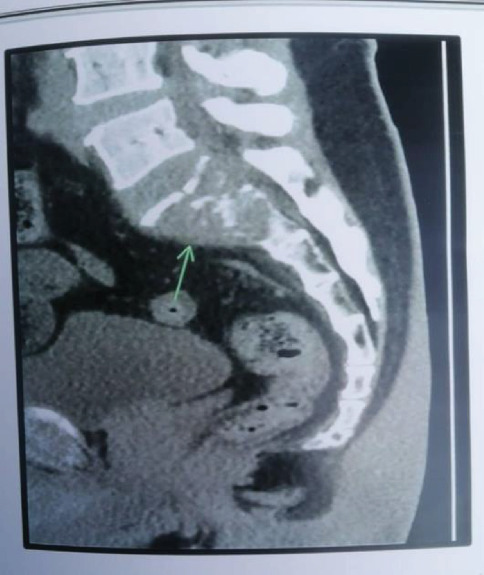
CT spine. S1–S2 destructive lesion.

CT spinal sacrum revealed a destructive lesion in the S1–S2 region (Figure 2).

**Figure 3:**
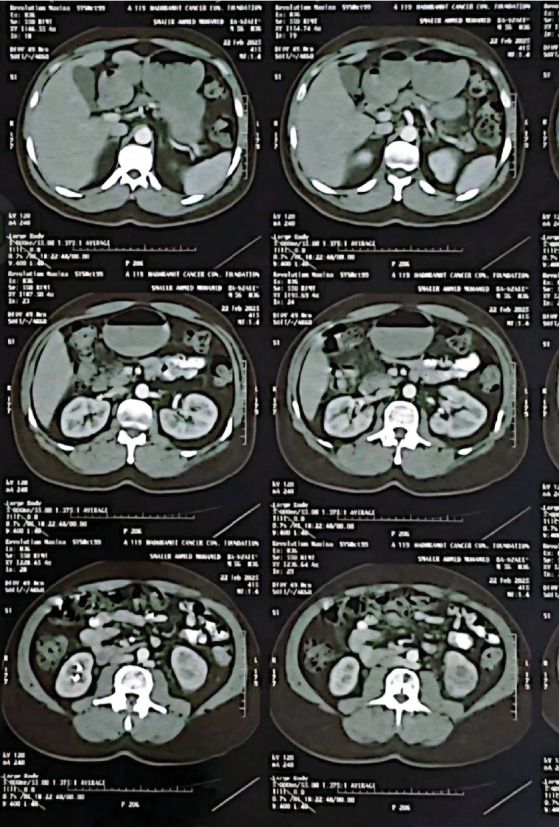
CT abdomen show left malignant lower pole renal mass.

CT abdominopelvic revealed 4 × 4.3-cm left lower pole renal tumor without lymph node. (Figure 3).

PET-CT confirmed isometabolic left renal lower pole solid mass with FDG uptake 4 SUV and S1–S2 destructive osseous single lesion with SUV max 5.6 mostly single Mets and no other metastases finding.

CT-guided biopsy from sacral mass showed nests of clear cells with positive PAX-8 and CA-IX staining, consistent with ccRCC.

• Treatment: Patient underwent partial left kidney lower pole nephrectomy with lymph node dissection and SBRT (45 Gy/15 fractions) on the sacrum, followed by sunitinib immunotherapy.
• Pathology report: Left partial nephrectomy, renal cell carcinoma clear type, free surgical margin, and nuclear Grade 2. Stage, pT1b N0.
• Outcome: Sacral pain resolution at 1 week post radiotherapy; stable disease on imaging after 3 months MRI and CT follow-up revealed clear operative bed and near disappear of S1–S2 destructive isolated sacral lesion.

## 3. Discussion

The presented case of a solitary sacral metastasis from ccRCC underscores both diagnostic and therapeutic challenges inherent in managing rare metastatic patterns. While bone metastases occur in approximately 30% of RCC patients, isolated sacral involvement remains exceedingly rare, accounting for < 1% of cases in reported series [6, 7]. This rarity often delays diagnosis, as sacral lesions may initially be attributed to trauma or degenerative conditions, as exemplified by this patient's presentation post-fall. The diagnostic pathway highlights the critical role of advanced imaging and histopathological confirmation. MRI and CT identified the lytic sacral lesion and primary renal tumor, while PET-CT excluded disseminated disease, a finding consistent with studies emphasizing PET-CT's utility in staging RCC [7].

### 3.1. Outcomes

The patient's favorable outcome symptomatic relief at 1 week and near resolution of the sacral lesion at 3 months demonstrates the potential of combined local and systemic therapies. Follow-up on 6 and 12 months revealed no recurrence.

## 4. Clinical Implications

This case reinforces that sacral metastasis, though rare, must be considered in RCC patients with unexplained back pain or neurologic symptoms. A low threshold for advanced imaging and biopsy is essential. Furthermore, multidisciplinary collaboration integrating surgery, radiation, and systemic therapy can achieve meaningful palliation and disease control, even in anatomically complex sites. Future studies should explore optimal sequencing of TKIs and radiotherapy to refine such protocols.

## 5. Conclusion

Isolated sacral metastases from ccRCC represent a diagnostic and therapeutic challenge due to their rarity and anatomic complexity. This case illustrates that timely imaging, histologic confirmation, and multimodal therapy can yield favorable short-term outcomes. Continued reporting of such cases is vital to augment the limited evidence guiding management of these rare metastases.

## Data Availability

Research data are not shared.
